# Novel *Wolbachia*-transinfected *Aedes aegypti* mosquitoes possess diverse fitness and vector competence phenotypes

**DOI:** 10.1371/journal.ppat.1006751

**Published:** 2017-12-07

**Authors:** Johanna E. Fraser, Jyotika Taneja De Bruyne, Iñaki Iturbe-Ormaetxe, Justin Stepnell, Rhiannon L. Burns, Heather A. Flores, Scott L. O’Neill

**Affiliations:** Institute of Vector-Borne Disease, Monash University, Clayton, VIC, Australia; University of California Riverside, UNITED STATES

## Abstract

*Wolbachia pipientis* from *Drosophila melanogaster* (*w*Mel) is an endosymbiotic bacterium that restricts transmission of human pathogenic flaviviruses and alphaviruses, including dengue, Zika, and chikungunya viruses, when introduced into the mosquito vector *Aedes aegypti*. To date, *w*Mel-infected *Ae*. *aegypti* have been released in field trials in 5 countries to evaluate the effectiveness of this strategy for disease control. Despite the success in establishing *w*Mel-infected mosquitoes in wild populations, and the well-characterized antiviral capabilities of *w*Mel, transinfecting different or additional *Wolbachia* strains into *Ae*. *aegypti* may improve disease impact, and perhaps more importantly, could provide a strategy to account for the possible evolution of resistant arboviruses. Here, we report the successful transinfection of *Ae*. *aegypti* with the *Wolbachia* strains *w*MelCS (*D*. *melanogaster*), *w*Ri (*D*. *simulans*) and *w*Pip (*Culex quinquefasciatus)* and assess the effects on *Ae*. *aegypti* fitness, cytoplasmic incompatibility, tissue tropism and pathogen blocking in a laboratory setting. The results demonstrate that *w*MelCS provides a similar degree of protection against dengue virus as *w*Mel following an infectious blood meal, and significantly reduces viral RNA levels beyond that of *w*Mel following a direct challenge with infectious virus in mosquitoes, with no additional fitness cost to the host. The protection provided by *w*Ri is markedly weaker than that of *w*MelCS, consistent with previous characterisations of these lines in *Drosophila*, while *w*Pip was found to substantially reduce the fitness of *Ae*. *aegypti*. Thus, we determine *w*MelCS as a key candidate for further testing in field-relevant fitness tests and viremic blood feeding challenges in a clinical setting to determine if it may represent an alternative *Wolbachia* strain with more desirable attributes than *w*Mel for future field testing.

## Author summary

Dengue viruses are transmitted by the *Aedes aegypti* mosquito, with an estimated 390 million human infections occurring per year worldwide. There is no approved antiviral therapeutic, and vaccines described so far have had limited efficacy. Recently, the endosymbiotic bacterium *Wolbachia* from *Drosophila melanogaster* (*w*Mel) has been used to infect *Ae*. *aegypti* populations as a novel technology for reducing dengue virus transmission. Here we report the generation of three new mosquito lines infected with the *Wolbachia* strains *w*MelCS, *w*Ri and *w*Pip. Each line induced cytoplasmic incompatibility and was effectively maternally transmitted, as required for rapid spread through uninfected mosquito populations. Each *Wolbachia* strain was also found to reside in the salivary glands; a key tissue involved in viral transmission. Perhaps most importantly, *w*MelCS inhibited dengue virus replication and dissemination in mosquitoes following an infectious blood meal or intrathoracic injection, providing a similar level of protection as that described for *w*Mel. *w*MelCS therefore warrants further investigation as a potential release strain in future field trials.

## Introduction

Arboviruses transmitted by the *Aedes aegypti* mosquito, including dengue (DENV), Zika (ZIKV) and chikungunya (CHIKV) are emerging threats that impose an increasing health burden on tropical and subtropical regions of the world. While these viruses usually cause self-limiting febrile disease, severe manifestations such as hemorrhagic shock can lead to death. In the absence of specific antiviral therapeutics and suboptimal vaccines [1–4], treatment is supportive only and limiting virus transmission has been largely dependent on vector control. The increasing global incidence of these diseases demonstrates the lack of effectiveness of current control programs and a need for novel efficacious and cost-effective alternatives [5].

In 2011, the first releases of *Ae*. *aegypti* mosquitoes carrying the artificially transinfected endosymbiotic bacterium *Wolbachia pipientis* began in field trials in Northern Australia as part of the Eliminate Dengue Program [6]. *Wolbachia* is a gram negative, obligate endosymbiont that is maternally transmitted and can impart antiviral properties to arthropod hosts. It is estimated that at least 40% of all terrestrial arthropod species are infected with *Wolbachia* [7] which can also manipulate host biology to induce feminization, parthenogenesis, cytoplasmic incompatibility (CI) and male-killing [8, 9]. Of these traits, CI is the most common, induced by many (but not all) *Wolbachia* strains, and enables the spread of *Wolbachia* into a new host population by providing females with *Wolbachia* an indirect reproductive advantage. In crosses between uninfected females and *Wolbachia*-infected males, CI results in the embryonic lethality of offspring. However, *Wolbachia*-infected females can mate with both uninfected and infected males successfully. CI, coupled with the maternal transmission (MT) of *Wolbachia* leads to the rapid invasion of the host population [8, 9].

The antiviral activity *Wolbachia* can provide to its host is a more recently described phenomenon [10]. The mechanism that drives this protection is not well understood but may involve priming of the insect innate immune response pathways in new *Wolbachia* infections, and competition for resources such as cholesterol [11–13].

Field trials to date have utilized *Ae*. *aegypti* transinfected with the *Drosophila melanogaster* native *Wolbachia* strain, *w*Mel, and a more pathogenic form, *w*MelPop-CLA, isolated from a lab stock of *D*. *melanogaster* [6, 14, 15]. While these strains have been shown to restrict replication and dissemination of several virus genera including flaviviruses and alphaviruses in *Ae*. *aegypti*, due to technical difficulties associated with introducing *Wolbachia* into a new species, the full potential of using alternative *Wolbachia* strains has remained largely unexplored.

This study set out to provide an initial examination of *Ae*. *aegypti* transinfected with novel *Wolbachia* strains to identify key candidate strains that could potentially contest, or even improve on the current pathogenic protection afforded to *Ae*. *aegypti* by *w*Mel, whilst avoiding fitness costs to the mosquito such as impaired egg viability and larval development as described for *w*MelPop-CLA infections [10, 16–19]. Given the cost and difficulty in taking alternative strains through a pipeline of more field-relevant laboratory testing and then into comparative field testing the goal of this work was to determine if any of the newly generated transinfected lines reported here could be removed from this pipeline in a first round of comparative testing.

For a *Wolbachia*-transinfected *Ae*. *aegypti* line to be considered for examination in further field-relevant experiments, the *Wolbachia* strain must provide strong protection against virus replication, demonstrate near-complete MT, and induce CI to enable rapid spread into the wild mosquito population, whilst inducing minimal fitness cost to the mosquito host.

To predict which *Wolbachia* strains may provide these traits we looked to past studies, most of which have been performed using natively *Wolbachia*-infected, artificially transinfected, or introgressed *Drosophila* lines, with the RNA viruses Drosophila C virus (DCV) or Flock House virus (FHV) as models. From these studies, we identified *w*MelCS (from *D*. *melanogaster*) and *w*Ri (from *D*. *simulans*) as key candidates [20–24].

Although *Ae*. *aegypti* mosquitoes do not naturally carry *Wolbachia*, a number of other mosquito species have natural infections including the closely related species *Aedes albopictus* (*w*AlbA and *w*AlbB) and *Aedes notoscriptus* (*w*Noto) as well as more distantly related species such as *Culex quinquefasciatus* (*w*Pip). *w*AlbB has previously been transinfected into *Ae*. *aegypti* and shown to provide strong protection against DENV while showing more resilience to heat stress but poorer larval competitive ability compared to *w*Mel [13, 25–29], indicating the difficulty in assessing strains for performance from relatively crude experimental studies and the need for comparative studies to be undertaken under field release conditions. *w*Pip has been reported to provide protection against West Nile virus (WNV) in its natural host, identifying this strain as a possible candidate for transinfection in *Ae*. *aegypti* [30].

Here we report the generation of three new *Ae*. *aegypti* lines, transinfected with *Wolbachia* strains *w*Ri, *w*MelCS and *w*Pip, and describe an initial relative evaluation of these strains, examining the effects on mosquito fitness, MT, CI, tissue tropism and DENV blocking. The findings reveal *w*MelCS as a potential candidate for future examination under more rigorous field-relevant testing and also indicate that *w*Ri and *w*Pip can be removed as candidates for future testing.

## Results

### Maternal transmission and cytoplasmic incompatibility

MT of a *Wolbachia* strain and its ability to induce CI are key features that must be conserved when considering new *Wolbachia*-infected *Ae*. *aegypti* lines for field releases. CI occurs when an uninfected female mated with a *Wolbachia*-infected male cannot produce viable offspring. This phenomenon ensures all offspring will be *Wolbachia*-positive, enabling effective spread of *Wolbachia* throughout a wild population.

MT was assayed by crossing *Wolbachia*-infected females (at least 5 generations removed from the G0 line; G5) with males from their respective *Wolbachia*-free tetracycline (Tet)-treated line and assaying for the presence of *Wolbachia* in adult progeny. The crosses revealed near-complete MT for *w*MelCS and *w*Pip (99.4 and 98.8%, respectively), similar to reported values for *w*Mel (100% with a 95% confidence interval lower boundary of 89%; [15]), while *w*Ri sustained some breakdown of MT at 87.5% (Table 1).

**Table 1:** Maternal transmission of *Wolbachia* assessed from progeny of crosses between infected females and uninfected males.

	No. Parent Females	No. Female progeny	No. *Wolbachia* positive (Maternal Transmission %)
*w*MelCS	37	651	647 (99.4%)
*w*Ri	54	1253	1096 (87.5%)
*w*Pip	49	434	429 (98.8%)

**Fig 1:**
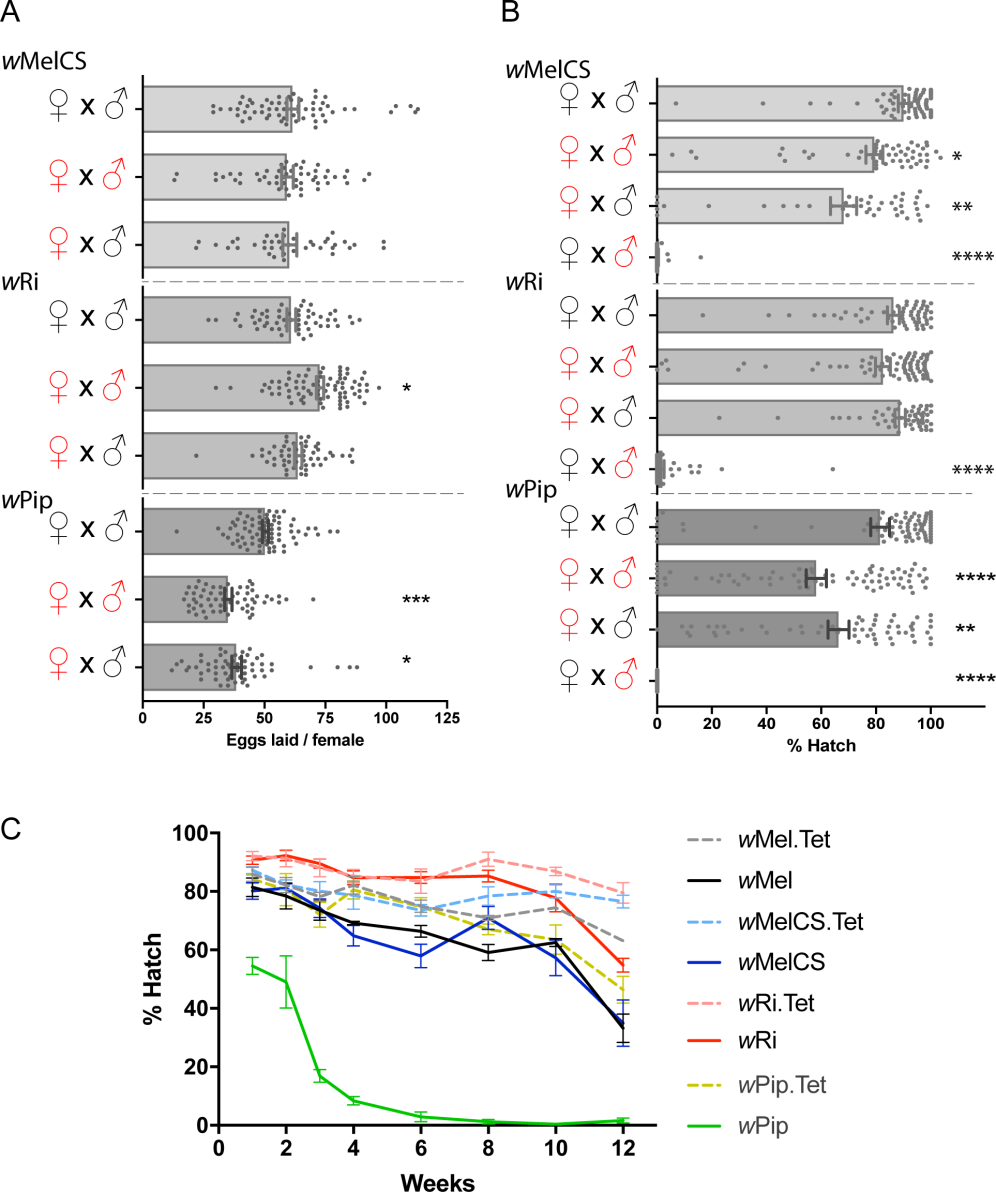
CI, fecundity and egg diapause viability in *w*MelCS, *w*Ri, and *w*Pip transinfected *Ae*. *aegypti*. (A) Fecundity was determined as a measure of eggs laid per female. Bars are the mean number of eggs laid ± SEM from >40 females (individual data points are superimposed). Symbols for tetracycline-treated mosquitoes (uninfected) are in black, *Wolbachia*-infected are in red. Asterisks indicate significance compared to Tet x Tet controls (Kruskal-Wallis, Dunn's test, * for <0.05, ** for <0.01, *** for <0.001, **** for <0.0001). (B) Hatch rates for crosses between infected and uninfected mosquitoes show CI and successful hatching. Symbol codes and statistics are as per (A). Data are the mean ± SEM from >40 females (individual data points are superimposed). (C) Eggs from gravid females were collected over 72 h, from 3-days post blood meal. Eggs were dried slowly over 3–5 days then stored in a humid, airtight container. Batches of 100–500 eggs were hatched after 1, 2, 3, 4, 6, 8, 10 and 12 weeks. Hatched larvae were counted at 2nd instar stage until no hatch was observed for a week then the percent hatch calculated. Statistical analysis was performed using 2way ANOVA Sidak's test (n = 4 at each time point). *w*Pip hatch rate was significantly reduced at all weeks compared to *w*Pip.Tet (p<0.0001). *w*Mel hatch rate was significantly reduced at weeks 4, 8, 10, (p<0.05) and 12 (p<0.0001) compared to *w*Mel.Tet. *w*MelCS had a significantly reduced hatch rate at weeks 10 and 12 compared to *w*MelCS.Tet (p<0.01 and p<0.0001, respectively). *w*Ri had a significantly reduced hatch rate at week 12 only, compared to *w*Ri.Tet (p<0.0001).

CI was determined for each line by measuring the hatch rate from crosses between Tet-treated females and infected males from each respective *Wolbachia*-infected line (Fig 1B). Control crosses where Tet-treated males and females were mated showed high hatch rates (>80%), while in CI crosses, near-complete sterility was observed in all three lines (0.3, 1.8 and 0% for *w*MelCS, *w*Ri, and *w*Pip, respectively), comparable to previous reports for *w*Mel (0%) [15, 26]. Note that newly established lines can display some variability between individuals which may explain the small differences in MT and CI shown here for *w*MelCS and *w*Pip.

### Analysis of mosquito fitness costs induced by each *Wolbachia* strain

#### Fecundity, hatch rate and egg diapause viability

Fecundity was measured as the number of eggs laid/female following mating between *Wolbachia*-infected males and females, and *Wolbachia*-infected females and Tet-treated males, compared to the control cross of Tet-treated males and females. For *w*Ri and *w*MelCS, the number of eggs laid/female did not significantly decrease irrespective of these matings, with the *w*Ri-infected male and female cross in fact showing a slight but significant (p<0.05 Kruskal-Wallis, Dunn's test) increase in eggs laid/female, indicative of a positive effect on fecundity (Fig 1A). For *w*Pip, relative to the Tet control cross, the mean number of eggs/female decreased for crosses between infected females and males, and infected females crossed with uninfected males (p<0.001, p<0.05, respectively), suggesting *w*Pip reduces fecundity for this line.

We next examined the egg hatch rates of each cross. No significant reduction in hatch rate was observed for *w*Ri crosses between *Wolbachia*-infected females and males, when compared to Tet-treated male and female crosses. *w*MelCS showed a slight although significant reduction in the hatch rate between *Wolbachia*-infected females and males, compared to the Tet-treated control cross (79% and 90%, respectively; p<0.05, Kruskal-Wallis, Dunn's test) (Fig 1B), similar to that reported for *w*Mel [15, 26]. Hatch rate was more dramatically impaired for *w*Pip, with rates dropping from 82% for the Tet-treated control cross, to 58% for *Wolbachia*-infected male and female crosses (p<0.0001). For *w*MelCS and *w*Pip, crosses between infected females and uninfected males also resulted in significant reductions in hatch rate (68 and 66%, compared to respective Tet control cross hatch rates of 90 and 82%, p<0.01), perhaps suggesting there is a small fitness cost for these strains of *Wolbachia* in eggs, although these reductions were similar to those described previously for *w*Mel [15].

To determine the effect of *Wolbachia* on egg viability and storage, eggs were collected and stored for 1–12 weeks before determining hatch rates. The benchmark transinfected line, *w*Mel had a slight, but significant reduction in hatch rate following 4, 8, and 10 weeks storage (p<0.05, 2way ANOVA Sidak's test) compared to its Tet control line, with a slightly larger reduction after 12 weeks (p<0.0001) (Fig 1C). *w*Ri and *w*MelCS lines displayed similar hatch rates (>60%) across all ages until week 10 (no significant difference in hatch rates compared to the respective Tet control lines until week 8). By contrast, *w*Pip eggs appeared to deteriorate rapidly, with < 20% hatch rate observed after 3 weeks of aging (significant reductions at all weeks tested, p<0.0001, compared to *w*Pip.Tet). Together, these data indicate a substantial fitness cost in the *w*Pip line.

#### Longevity

**Fig 2:**
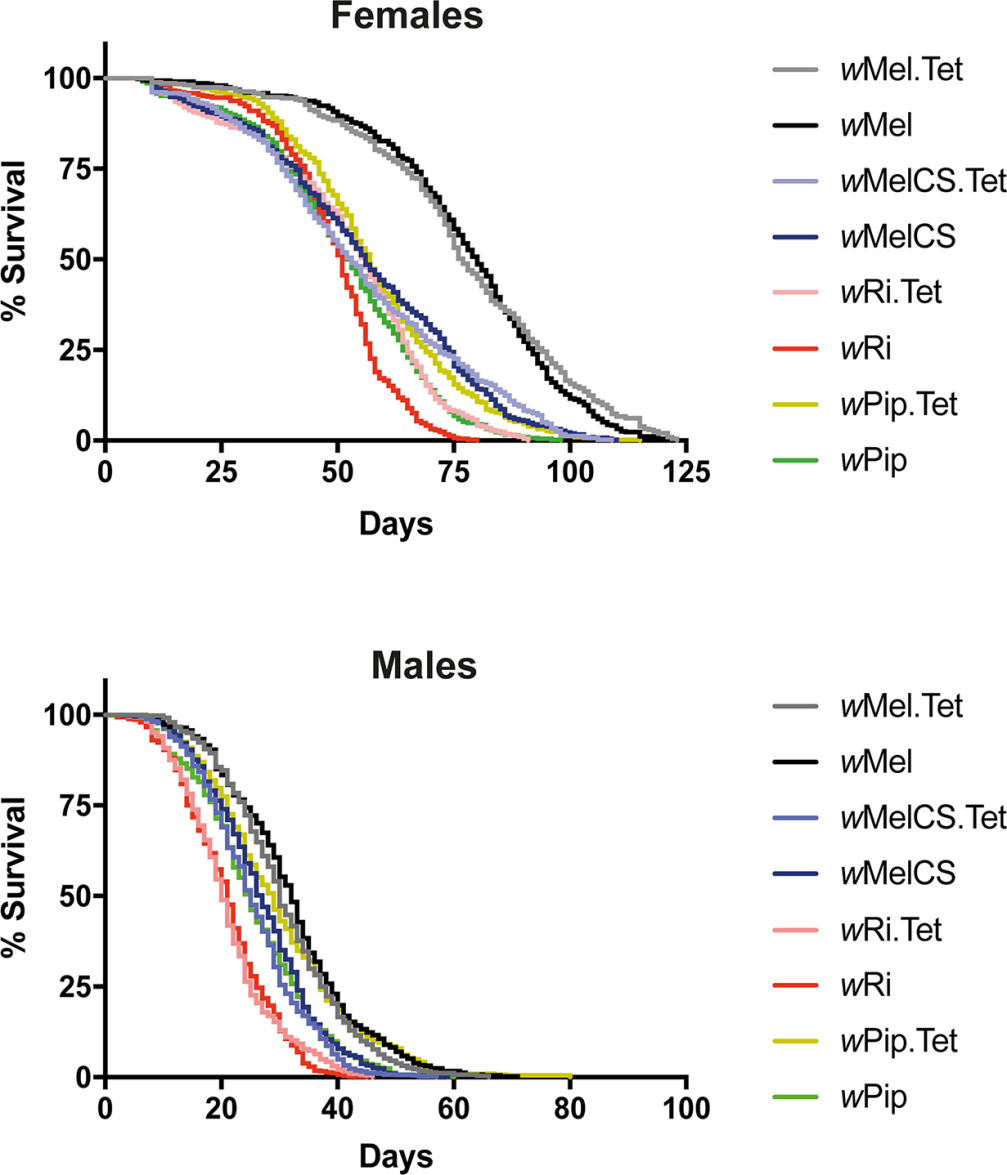
Longevity in transinfected *Ae*. *aegypti* lines. Triplicate cages of age-controlled (emergence within 24 h) adults (~150 males and ~150 females/cage) were maintained at 26°C, 65% relative humidity and a 12:12 h light:dark cycle in a climate controlled room. The number of dead males and/or females was recorded and carcasses removed daily until all mosquitoes in the cages were dead. Data are the total % survival from the three cages/line. Significant differences were observed for *w*Ri females (p<0.0001), *w*Mel (p<0.05) and *w*MelCS (p<0.05) males, relative to their respective Tet control line. *w*Pip had a significantly shorter lifespan for both males and females compared its matched Tet-control (p<0.0001). Statistical analysis was performed using a Log-rank (Mantel-Cox) test.

Effects on the survival of mosquitoes from each line were next examined over time (Fig 2). *w*Mel, *w*MelCS and *w*Ri *Wolbachia*-infected females tracked closely with their Tet-treated controls suggesting the infection had minimal influence on longevity. Similar relative survival rates were observed for males. Minor, but significant differences were observed for *w*Ri females (p<0.0001, Log-rank (Mantel-Cox) test) and *w*Mel (p<0.05) and *w*MelCS (p<0.05) males, relative to their respective Tet control lines. *w*Pip was the only line that showed a substantially shorter lifespan for both males and females compared to the Tet-control (p<0.0001). Since <1% of wild female *Ae*. *aegypti* are expected to live beyond 3 weeks [31], it’s expected that no infection described here would likely impact on the lifespan of the mosquito in the field.

We also examined compatibility between *w*Mel and *w*MelCS, since *w*MelCS-infected *Ae*. *aegypti* appeared to be the least compromised line from these initial screening experiments. We found these strains to be bidirectionally compatible with crosses between *w*MelCS females and *w*Mel males, and *w*Mel females and *w*MelCS males, each resulting in successful hatch rates (71 and 62%, respectively, S1 Fig). These data suggest that *w*MelCS could not be released over an existing *w*Mel strain and expect to overtake *w*Mel by CI alone. The ability of either of these strains to replace another in a field setting will be dependent upon the which strain is least costly to host fitness.

#### *Wolbachia* density and distribution

**Fig 3:**
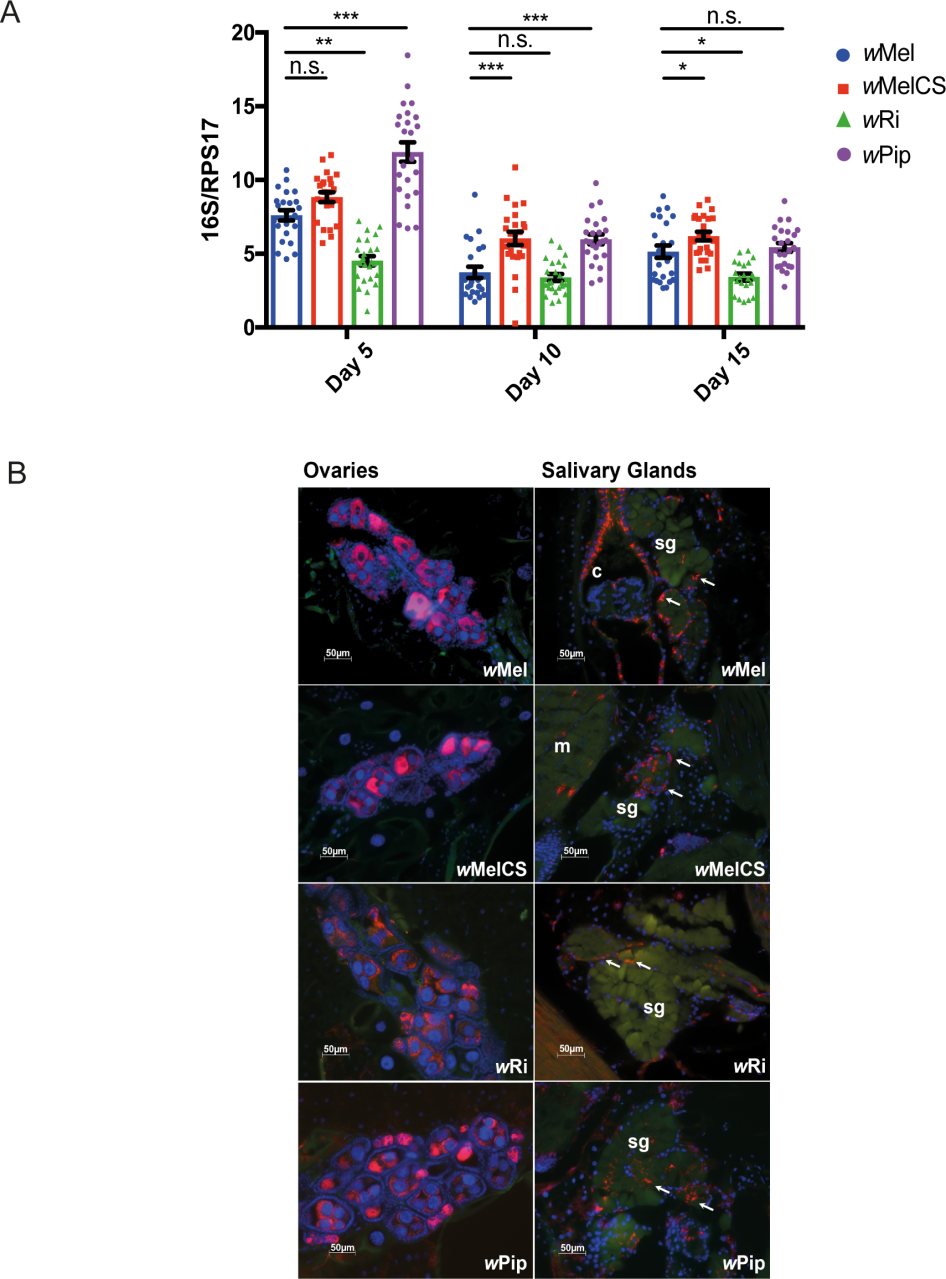
*Wolbachia* density and distribution in transinfected *Ae*. *aegypti* lines. (A) Density of *Wolbachia* within 5-, 10- and 15-day old whole female mosquitoes was determined by qPCR using primers directed to the conserved *16S* rRNA gene. Density is expressed as the mean ratio between *16S* and the *Ae*. *aegypti* host *rps17 gene*. Data are the mean and SEM of 24 mosquitoes. Asterisks indicate significance compared to *w*Mel at each time point (Kruskal-Wallis, Dunn's test with multiple test corrections; *p<0.05, **p<0.01, ***p<0.001, n.s. not significant). (B) The distribution of *w*MelCS, *w*Ri and *w*Pip *Wolbachia* strains in mosquitoes was determined in sections of paraffin-embedded female mosquitoes (5 to 7-day old) using fluorescence *in situ* hybridisation (FISH). The fluorescently labelled 16S probe detects the *16S* rRNA gene from all four *Wolbachia* strains. Total DNA was stained in blue using DAPI and a green filter was included to increase contrast with surrounding tissues. *Sg* indicates salivary gland tissue, *m* indicates muscle, and *c* indicates cardia. White arrows identify select regions of *Wolbachia* staining.

Total *Wolbachia* density was measured in 5-, 10- and 15-day old adult females of each line (at least G9) and compared to the benchmark line *w*Mel at each time point, using qPCR with primers specific to the highly conserved *Wolbachia* ribosomal RNA gene, *16S*, and *Ae*. *aegypti rps17* gene to normalize for DNA input. Overall, the density of each strain reduced as the mosquitoes aged (Fig 3A). *w*MelCS density was significantly higher than *w*Mel at days 10 and 15 post emergence (p<0.001, p<0.05, Mann-Whitney test), as was *w*Pip at days 5 and 10 (p<0.001). *w*Ri density was nearly half that of *w*Mel at day 5 and was significantly reduced at day 15 (p<0.01 and p<0.05, respectively), but not significantly different at day 10. Despite these variations, all densities measured were within a 1log_10_ range of each other, indicating each *Wolbachia* strain maintains itself at high density.

The localization of *Wolbachia* within ovary and salivary gland tissues was next examined in 5–7 day old adult female mosquitoes using fluorescence *in situ* hybridization (FISH). Mosquitoes were formaldehyde-fixed, and paraffin-embedded tissue sections stained using a probe specific for *Wolbachia 16S* gene (red, Fig 3B). Total DNA was stained with DAPI (blue). Consistent with the strong MT shown in Table 1 for all lines, each strain was abundant in ovarian tissues, with distribution comparable with *w*Mel (Fig 3B). Similar distribution of *Wolbachia* was also observed for all lines in the salivary glands, a key tissue involved in viral transmission.

#### Restriction of DENV-3 replication by *w*Ri and *w*MelCS in *Ae. aegypti*

**Fig 4:**
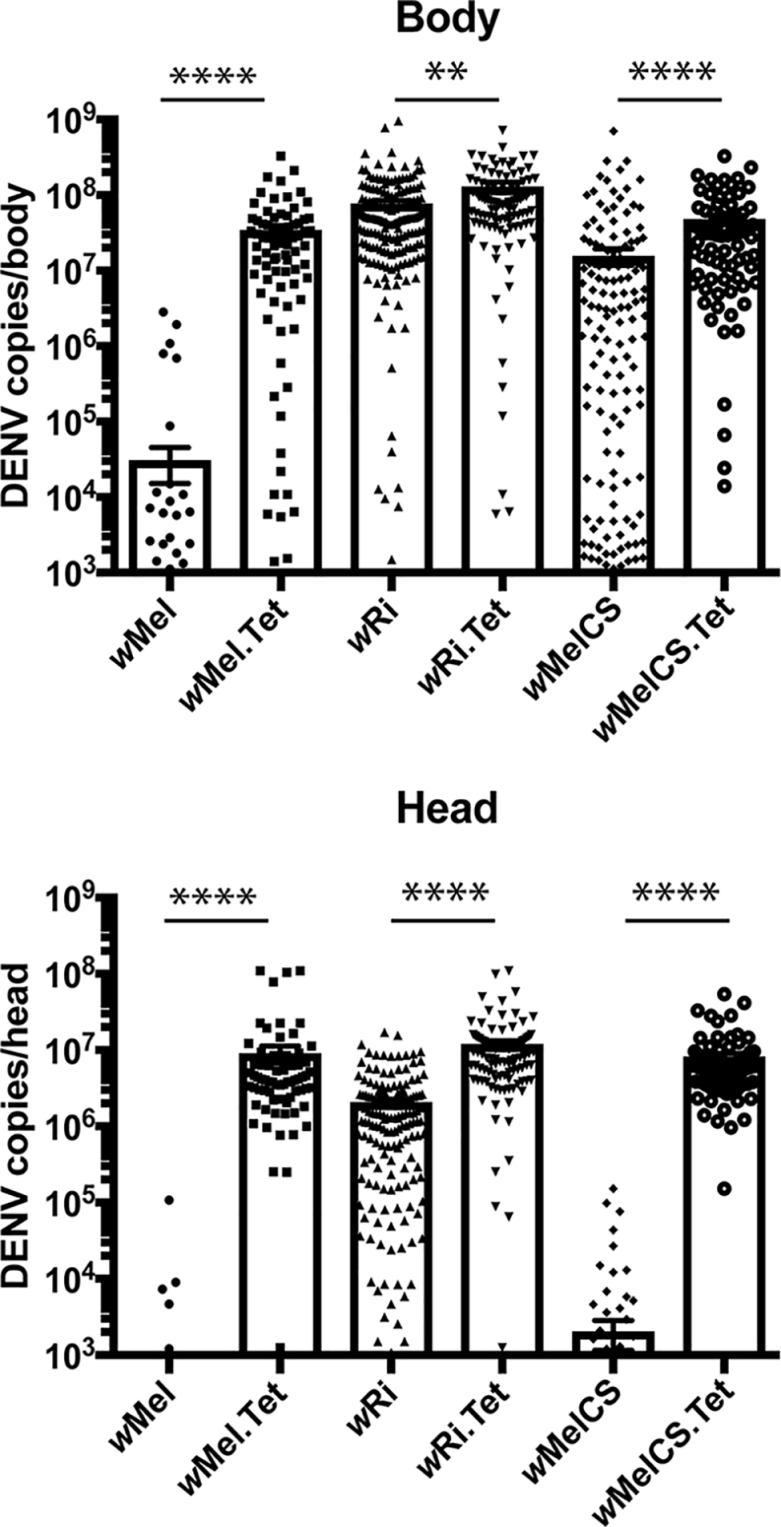
*w*MelCS and *w*Ri *Wolbachia* strains inhibit DENV-3 replication and dissemination following an infectious blood meal. Seven-day old female mosquitoes were fed a blood meal of fresh DENV-3 (2.0 x 10^6^ TCID_50_/ml) mixed 1:1 with sheep blood (n = 500 per *Wolbachia*-infected line, n = 200 per respective Tet control line). Mosquitoes were sorted immediately to identify those that took a blood meal, and incubated for 14 days. Mosquito heads were then separated from the thorax and total RNA was extracted from each head and remaining mosquito body. DENV genome copies were determined by qRT-PCR for each body as a measure of infection, and for each head as a measure of viral dissemination. Data are the mean genome copies per mosquito body (top) or head (bottom) ± SEM, and are representative of 3 independent experiments. Statistical analyses were performed using a Mann-Whitney test where ** p < 0.01, ****p<0.0001.

To compare the viral-blocking capacity of these lines, 7 day old mosquitoes were fed an infectious blood meal containing 2.0 x 10^**6**^ TCID_50_/ml (DENV-3), then incubated for 14 days at 26°C. Due to the reduced fitness of *w*Pip mosquitoes we elected to exclude this line. Vector competence analyses were performed on *w*Ri and *w*MelCS lines, and compared to *w*Mel. Mosquitoes were collected, the head separated from each mosquito body, then total RNA extracted to measure rates of infection (as determined by bodies positive for DENV RNA) and dissemination (heads positive for DENV RNA). Consistent with previous findings, *w*Mel reduced the mean DENV RNA levels by 3log_10_ in mosquito bodies (3 x 10^4^ copies/body compared to 3 x 10^7^ copies/body in the relevant Tet control line, p<0.0001, Mann-Whitney test), as well as reducing the infection rate, with just 9% of *w*Mel mosquito bodies scoring positive for DENV infection compared to 94% in the matched Tet control line (where positive was defined as >1000 copies/body, see Materials & Methods; Table 2 with the same data used to generate Fig 4). By contrast, *w*Ri caused only a slight, although significant (p<0.01) reduction in viral copies (7.6 x 10^7^ copies/body compared to 1.3 x 10^8^ copies/body for the matched Tet control line) with no reduction in infection rate (96% compared to 98%, respectively). *w*MelCS gave a phenotype intermediate of *w*Mel and *w*Ri, with a wide spread in the number of DENV copies/body observed, with a mean of 1.5 x 10^7^ RNA copies compared to 4.7 x 10^7^ RNA copies for its matched Tet control line (p<0.0001). Interestingly, DENV-3 dissemination, key for viral transmission, was similarly restricted between *w*Mel and *w*MelCS lines, with ~4log_10_ reduction in viral RNA copies determined in the head of each line relative to the matched Tet control, and just 2 and 9% infection rates, respectively, compared to 83 and 96% for the Tet controls. While the mean DENV RNA copies/head of *w*Ri-infected mosquitoes was significantly reduced (2 x 10^6^ copies compared to 9.5 x 10^6^ copies for the Tet control line), the dissemination rates were not reduced (94% compared to 95%, respectively). Together, these results indicate that *w*Ri shows weaker DENV-blocking capacity while the ability of *w*MelCS to restrict viral dissemination appears to be similar to that of *w*Mel.

**Table 2:** Restriction of DENV-3 infection and dissemination by *Wolbachia* strains *w*Ri and *w*MelCS.

Line	Body		Head	
	Number DENV + (total screened)	% DENV +^#^	Number DENV + (total screened)	% DENV +^#^
*w*Mel	22 (250)	9	5 (250)	2
*w*Mel.Tet	78 (83)	94	69 (83)	83
*w*MelCS	135 (240)	56	21 (240)	9
*w*MelCS.Tet	74 (76)	97	73 (76)	96
*w*Ri	166 (169)	98	159 (169)	94
*w*Ri.Tet	103 (107)	96	102 (107)	95

# Calculated as a percentage of total engorged mosquitoes

**Fig 5:**
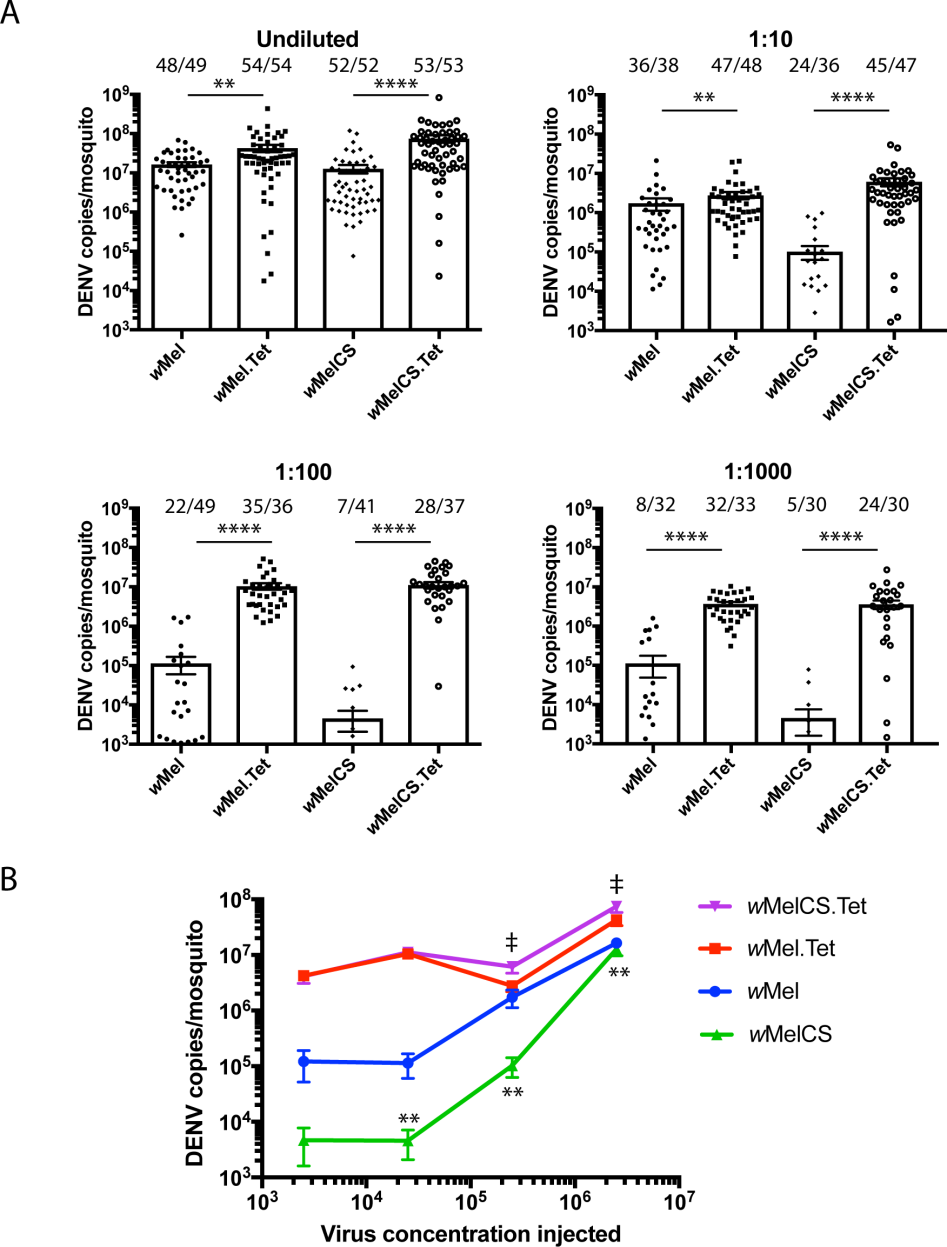
*w*MelCS provides superior blocking of DENV-3 genome replication in a mosquito injection challenge model. (A) DENV-3 was injected into the thorax of 6 or 7-day old female mosquitoes at 2.5 x10^6^ TCID_50_/ml (undiluted) or 10, 100 or 1000-fold dilutions thereof. RNA was extracted from whole mosquito bodies 7-days post infection and virus replication was quantified by qRT-PCR. Data are the mean number of genome copies per mosquito ± SEM. Number of DENV-3 positive mosquitoes/total *n* are indicated above each bar. ** p < 0.01, ****p<0.0001, Mann-Whitney test. (B) The mean DENV genome copies and SEM from (A) are replotted as a function of virus concentration injected. Significant differences in the mean RNA copies of *w*Mel and *w*MelCS lines are indicated by ** (p<0.01). Significant differences in the mean RNA copies of *w*Mel.Tet and *w*MelCS.Tet lines are indicated by **‡** (p<0.05), Mann-Whitney test. Injection dilutions were performed as independent experiments, with the data combined to produce the final data series.

In order to more closely examine the blocking capacity of *w*MelCS we next used an injection challenge model which bypasses the midgut barrier leading to a higher level of tissue infection, to directly compare viral restriction in *w*Mel and *w*MelCS lines. Female mosquitoes (7 days old) were injected with 2.5 x 10^6^ TCID_50_/ml of DENV-3, or 10-fold dilutions thereof. Mosquitoes were incubated for 7 days before total RNA extraction from entire mosquitoes. Note that DENV-injected mosquitoes have virus delivered directly to the thorax, bypassing the midgut barrier to infection, therefore measuring dissemination rates in these mosquitoes is not a relevant test. When mosquitoes were injected with undiluted virus, both *Wolbachia* lines struggled to restrict viral replication with the number of DENV copies reduced by < 1log_10_ relative to each matched Tet control line (Fig 5A and 5B). When mosquitoes were injected with 2.5 x 10^5^ TCID_50_/ml (1:10), *w*MelCS reduced DENV RNA copies by ~2log_10_ compared to <1log_10_ for *w*Mel, indicating that *w*MelCS may be more effective at restricting replication of DENV-3 than *w*Mel when challenged with a highly infectious setting. This trend continued as the virus was diluted further, with significantly lower viral RNA levels measured in *w*MelCS mosquitoes when injected with 2.5 x 10^4^ TCID_50_/ml (1:100) (p<0.01, Mann-Whitney test) and substantially (although not significantly) reduced RNA levels at 2.5 x 10^3^ TCID_50_/ml (1:1000). Importantly, markedly lower rates of infection were also observed in *w*MelCS compared to *w*Mel-infected mosquitoes for the three lowest injected virus concentrations (“number of DENV-positive mosquitoes/total injected mosquitoes” are indicated above each bar). Together, the laboratory-based infectious blood feeding and injection challenge systems provide a relative, initial evaluation of the vector competence of these new lines, and identify *w*MelCS as a potential candidate to progress to more intensive human viremic blood challenge experiments.

## Discussion

Field releases of *w*Mel-transinfected *Ae*. *aegypti* in order to establish *Wolbachia* in wild populations have been expanding over the past 6 years in multiple countries and the technology shows significant potential to have a large impact on arbovirus disease. However, there is still a need to further develop and optimize the technology to maximize its impact and to that end testing alternative strains of *Wolbachia* for greater field effectiveness is a priority. To most effectively measure performance, strains need to be compared in field release experiments. These tests are extremely involved, expensive and time consuming and as a result we have used a panel of laboratory-based tests to determine if a suite of newly developed strains show sufficient promise to be progressed through a development pipeline to final field comparison.

Here we report for the first time the transinfection of *Ae*. *aegypti* with *Wolbachia* strains *w*MelCS, *w*Ri, and *w*Pip. *w*MelCS-infection had minimal effect on mosquito fitness, similar to *w*Mel. However, *w*Ri displayed reduced MT, and the introduction of *w*Pip lead to poor egg viability after short storage times, and weaker fecundity and hatch rates; traits that would be likely to compromise effective release strategies when performed in a field setting. Interestingly, *Ae*. *albopictus* transinfected with *w*Pip showed similar reductions in female fecundity and egg hatch rate [32], suggesting that *w*Pip may broadly induce this fitness cost when introduced into a non-native host. Although this may be context dependent, as work from Zhang *et al*. (2015) determined no fitness cost to *Ae*. *albopictus* when *w*Pip was introduced into mosquitoes that also contained the native *w*AlbA and *w*AlbB strains [33].

When tested for their ability to block DENV, *w*MelCS and *w*Ri restricted replication and dissemination following an infectious blood meal, although *w*Ri blocking was substantially reduced compared to *w*MelCS, with *w*MelCS restricting DENV dissemination to the mosquito head to a similar extent as *w*Mel. In a virus injection challenge model where mosquitoes are essentially overloaded with virus, *w*MelCS demonstrated superior blocking under all concentrations of virus tested. These findings indicate the blocking provided by *w*MelCS is robust, and suggests this line is worthy of further consideration in more exhaustive human viremic blood feeding experiments. While these future experiments are both costly and time consuming, they will provide a critical evaluation of vector competence, more likely to closely represent the performance of these mosquitos in the field, and may determine whether *w*MelCS should be progressed to field release comparisons [26, 34].

It has been inferred that the degree of blocking correlates with density of the *Wolbachia* strain in key tissues [35–37]. We show here that overall *Wolbachia* densities are only slightly higher in *w*MelCS-transinfected mosquitoes compared to *w*Mel, consistent with the similar virus-blocking phenotypes. In *w*Ri-containing mosquitoes, *Wolbachia* was generally found to be present at a lower density than *w*Mel, and was significantly worse at blocking DENV-3 replication and dissemination. Interestingly, when Osborne *et al*. (2009) examined blocking of DCV by *w*Ri and transinfected-*w*Mel in *D*. *simulans* they observed almost identical densities of *w*Ri and *w*Mel, similar protection against virus-induced mortality, yet *w*Ri did not reduce the viral load [20]. While this supports a role for utilizing *Wolbachia* densities to predict viral protection in mosquitoes, it highlights the complex nature of this tripartite interaction. Tissue specific quantitation of *Wolbachia* may provide further insight into the importance of *Wolbachia*-localization and density in virus blocking by each strain.

Given the substantial time it takes to introduce a new *Wolbachia* strain into *Ae*. *aegypti*, understanding how that strain is likely to behave, and the extent of pathogen protection it will provide once introduced into *Ae*. *aegypti*, will be of great benefit for ensuring effective transinfected lines are generated. Here, the increased DENV protection provided to *w*MelCS-infected mosquitoes compared to *w*Mel under certain infection conditions, is in line with work performed in *D*. *melanogaster* and *D*. *simulans* using DCV and FHV [22, 23]. Similarly, *w*Ri provided incomplete protection against these viruses in *D*. *simulans* [20], indicating that *Wolbachia*-infected *Drosophila* lines may be useful predictors for pathogen blocking in *Wolbachia*-infected mosquitoes. Further analyses of *Ae*. *aegypti* lines transinfected with different *Drosophila*-derived *Wolbacha* strains is required to determine if this trend holds. It should also be acknowledged that the characterisations described here were not repeated with independently generated lines, and there may be some variation between individual lines, depending on the genome and mitochondrial composition.

With a strong virus blocking phenotype, *w*MelCS may prove to be an effective candidate for field trials without displaying the fitness costs that prevented field establishment of *w*MelPop-CLA [18]. To test this prediction these mosquitoes now need to be examined in more realistic challenge settings, such as blood feeding on viremic patients.

## Materials and methods

### Mosquito rearing

All *Ae*. *aegypti* mosquitoes were reared and maintained as described previously [16, 26]. Adult mosquitoes were maintained at 26°C, 65% relative humidity (RH) and a 12 h light:dark cycle in a climate-controlled room. Mosquitoes were blood fed on the arms of human volunteers (Monash University human ethics permit CF11/0766-2011000387). The *Wolbachia*-infected *w*Mel line included in our experiments has been described previously [15, 38]. We generated uninfected lines for each strain by treatment of the infected lines with the antibiotic tetracycline (Tet) as described earlier [16, 39]. Briefly, *w*MelCS, wRi and wPip adults were fed on 10% sucrose solution containing 1 mg/ml Tet right after emergence for two generations followed by a recovery period of another two generations, whereby larvae rearing water from the infected lines was added to the respective Tet-treated lines to homogenize their gut flora. Tet-lines were reared for a further minimum of 2 generations before use in maternal transmission, CI, fitness, and vector competence experiments.

To exclude any influence of age and quality of eggs used in experiments, 5-day old females from all lines were fed by the same volunteer in one sitting. The eggs collected from these females were then dried and used as experimental material within 2–3 weeks. For experiments using the *w*Pip line, freshly laid eggs were used due to poor hatch rates following egg storage.

To exclude any influence of mosquito age on our experiments, age-controlled adults emerging within a 24 h window were used [40].

### *Wolbachia* transinfection

The *Wolbachia* uninfected and inbred PGYP1.Tet line was used as the recipient line for transinfection. *Wolbachia* strains were not cell line adapted as described previously [16], but were instead directly isolated from donor insects and introduced into *Ae*. *aegypti* embryos by cytoplasmic transfer. *w*MelCS was sourced from *Drosophila melanogaster* Canton S strain [41], *w*Ri was sourced from *Drosophila simulans* DSR strain (Riverside) [42], *w*Pip was isolated from *Culex quinquefasciatus* [43] (a kind gift from Karen Williams, Westmead Hospital, Australia). Embryonic microinjection, isofemale line establishment and selection for stably-infected lines were done as previously described [15, 16] with a few modifications. In brief, the *w*MelCS, *w*Ri and *w*Pip strains were purified from their respective donor insects, and microinjected into the posterior-pole of pre-blastoderm embryos of the recipient PGYP1.tet using methodology previously described [15, 16]. Surviving G0 adult females from microinjection were mated to PGYP1.tet males, blood fed and set up for oviposition as isofemales. G0 females that laid fertile eggs were screened using quantitative PCR (qPCR) as described below. Once the newly introduced *Wolbachia* strain was at 100% frequency in the population and maintained itself for at least 2 consecutive generations the line was considered stable. The *w*MelCS and *w*Ri lines were stable at G3 and *w*Pip at G5.

### Cytoplasmic incompatibility and maternal transmission

To investigate the level of *Wolbachia*-induced cytoplasmic incompatibility (CI) we set up paired crosses between *Wolbachia*-infected and Tet-treated *Ae*. *aegypti*. For each *Wolbachia* strain four crosses were set up: (1) a CI cross (Tet females x infected males), (2) a maternal transmission (MT) cross (infected females x Tet males), (3) an uninfected control cross (Tet females x Tet males) and (4) an infected control cross (infected females x infected males). Each cross consisted of a group of 70 virgin males and 70 virgin females and each group was allowed to mate for 5 days after emergence. On day five all females were blood fed by one human volunteer. Gravid females were aspirated into individual tubes three-days post blood feeding and allowed to oviposit on wet filter paper [26]. Three-days post oviposition female mosquitoes were removed and tested for presence of *Wolbachia* by qPCR using strain-specific primers as described in the PCR section.

Any paper containing less than 15 eggs was discarded. The egg papers from the remaining females were photographed and eggs were counted manually. The papers were then submerged in hatching water. Two days later the number of hatched larvae (2nd instar) was counted. Newly hatched larvae were counted daily until no hatching was recorded for three consecutive days. Any unhatched eggs were dried briefly for 48–72 h, hatched and counted again to get the maximum hatch.

To quantify the success of MT of *Wolbachia* to the next generation, hatched larvae from the MT cross (infected females x Tet males) were reared to adults and screened by qPCR for the presence of *Wolbachia*.

### Fitness determinants

#### Egg diapause viability

To assess the viability of eggs stored over time, age-controlled adults (emergence within 24 h; 100 males and 100 females) were placed in quadruplet 30x20x20cm cages. One cage of each line was blood-fed by the same human volunteer, to control for the potential variation caused by blood-meal quality and composition. This was repeated 4 times with 4 different volunteers. Female mosquitoes were given a blood meal at 5 days old. Three-days post blood meal, 12 oviposition cups were placed in each cage for three days. Approximately 72 h post oviposition, egg cups were removed, dried slowly over 3–5 days then stored in Whirlpak bags (Sigma) in an airtight container with saturated KCl solution to maintain humidity [16]. Batches of 100–500 eggs were photographed and counted, then hatched after 1, 2, 3, 4, 6, 8, 10 and 12 weeks from each cage. Hatched larvae were counted at 2nd instar stage until no hatch was observed for a week then the percent hatch calculated.

#### Fecundity and hatch rate

This was performed using the parameters described for CI and MT above. Fecundity was calculated as the average number of eggs laid/female, and hatch rate determined as the number of 2^nd^ instar larvae per total eggs hatched.

#### Adult longevity

Age-controlled adults (approximately 150 males and 150 females) were placed in triplicate 30x30x30cm cages. Mosquitoes were provided fresh 10% sucrose twice/week and maintained at 26°C, 65% RH and a 12:12 h light:dark cycle in a climate-controlled room. The number of dead males and/or females was recorded and removed daily until all mosquitoes in the cages were dead.

#### *Wolbachia* detection by PCR

The presence or absence of *Wolbachia* in transinfected mosquitoes was confirmed by qPCR using strain-specific primers. *w*Pip was detected by amplifying an *IS2* transposon, with 49 identical copies of this sequence in the *w*Pip genome (forward primer: 5’-GCACTTACCCTAACCAAAGGTAAC-3’, reverse primer: 5’ CTAACTTTAGGCCTCTATCGAAGAG-3’), *w*Ri was detected by amplifying a part of the gene *WRi_009390*, which encodes a hypothetical protein (forward primer: 5’CATGCCAATAACGAAATAGC -3’, reverse primer: 5’-TAGCAACTTTTCTTGCGAAC-3’). A combination of two primer sets was used to differentiate *w*MelCS from *w*Mel strains. One of the primers sets binds to *w*Mel and *w*MelCS in the *WD0513* region of the genome [34, 44] (forward primer: 5’CAAATTGCTCTTGTCCTGTGG-3’, reverse primer: 5’-GGGTGTTAAGCAGAGTTACGG-3’), Probe Cy5 TGAAATGGAAAAATTGGCGAGGTGTAGG- BHQ3. The second primer set binds to the polymorphic insertion sites of *w*MelCS at loci *IS5-WD1310* (forward primer: 5’- CTCATCTTTACCCCGTACTAAAATTTC-3’, reverse primer: 5’-TCTTCCTCATTAAGAACCTCTATCTTG-3’), Probe HEX TAGCCTTTTACTTGTTTCCGGACAACCT-BHQ1. For each sample, qPCR was performed using a LightCycler 480 II Instrument (Roche) using LightCycler 480 SYBR Green I Master (Roche) for *w*Pip and *w*Ri amplification and Taqman Probes Master for amplifying *w*MelCS according to the manufacturer’s protocol.

#### *Wolbachia* density and distribution

The density of *Wolbachia* in the three newly introduced strains, *w*MelCS, *w*Ri and *w*Pip was compared to the density in the long established *w*Mel strain [15, 16]. Total relative *Wolbachia* densities for the four lines were determined in whole, female mosquitoes at 5-, 10- or 15-days post emergence, using qPCR with primers to amplify a fragment of the gene coding *16S* rRNA (16S) (forward primer: 5’-GAGTGAAGAAGGCCTTTGGG-3’, reverse primer: 5’- CACGGAGTTAGCCAGGACTTC-3’, probe 5’ LC640-CTGTGAGTACCGTCATTATCTTCCTCACT-IowaBlackRQ-3’) and the reference *Ae*. *aegypti rps17* gene (forward primer: 5’-TCCGTGGT ATCTCCATCAAGCT-3’, reverse primer: 5’-CACTTCCGGCACGTAGTTGTC-3’, probe 5’FAM- CAGGAGGAGGAACGTGAGCGCAG-BHQ1-3’). *Wolbachia* densities were quantified relative to *rps17* using the delta CT method (2^CT^(reference)/ 2^CT^(target)).

The distribution of *w*MelCS, *w*Ri and *w*Pip *Wolbachia* in mosquitoes was determined in sections of paraffin-embedded female mosquitoes (5 to 7 days old) using fluorescence *in situ* hybridisation (FISH), as described in Moreira *et al*. (2009) [10]. The fluorescently labelled probes used can detect the *16S* rRNA from all three *Wolbachia* strains. Total DNA was stained in blue using DAPI and a green filter was included to increase contrast with surrounding tissues.

#### Vector competence

DENV-3 Cairns 08/09 strain stocks (Genbank accession number: JN406515.1) were prepared by inoculation of C6/36 cells with a multiplicity of infection (MOI) of 0.1 and collection of culture supernatant 6–7 days later. Virus concentrations were determined by TCID_50_ as previously described [45] using monoclonal antibody 4G2 [46].

For feeding experiments with DENV-3 (Cairns 08/09) infected blood, 100 seven-day old age-controlled female mosquitoes were placed in 500 mL plastic containers (five containers per *Wolbachia* line, two containers per Tet line), starved for up to 24 h and allowed to feed on a 50:50 mixture of defibrinated sheep blood and tissue culture supernatant containing freshly harvested 2.0 x 10^6^ TCID_50_/mL of DENV-3. Feeding was done through a piece of desalted porcine intestine stretched over a water-jacketed membrane feeding apparatus preheated to 37°C. Mosquitoes were left to feed in the dark for approximately 80 min. Fully engorged mosquitoes were placed in 500 mL containers at a density of < 20/container, and incubated for 14 d at 26°C with 65% RH and a 12 h light/dark cycle.

For adult microinjections, 60 six or seven-day old age-controlled female mosquitoes were anesthetized by CO_2_. The mosquitoes were injected intrathoracically with 69 nL of DENV-3 Cairns 08/09 strain, 2.5 x10^6^ TCID_50_/ml (or 10, 100 or 1000-fold dilutions thereof) in RPMI media (Life Technologies) using a pulled-glass capillary and a handheld microinjector (Nanoject II, Drummond Scientific). Injected mosquitoes were incubated for 7 days (10 mosquitoes/cup) at 26°C with 65% RH and a 12 h light/dark cycle. To quantify DENV-3 genomic copies, total RNA was isolated from DENV-3 mosquitoes (entire mosquitoes for injection experiments, or head and bodies separately for blood fed mosquitoes) using the RNeasy 96 QIAcube HT kit (Qiagen). DENV-3 RNA was amplified by qRT-PCR (LightCycler Multiplex RNA Virus Master, Roche), using primers to the conserved 3’UTR: Forward 5’-AAGGACTAGAGGTTAGAGGAGACCC; Reverse 5’- CGTTCTGTGCCTGGAATGATG; Probe 5’-HEX- AACAGCATATTGACGCTGGGAGAGACCAGA-BHQ1-3’and absolute copies determined by extrapolation from an internal standard curve generated from plasmid DNA encoding the conserved 3’UTR sequence. Mosquito extracts with ≥1000 copies of DENV per body were scored positive, based on the LOD_95_ (limit of detection 95%) for DENV-3 with this primer set.

### Data availability

All raw data are available within S1 Data.

## Ethics statement

Blood feeding by volunteers (Monash University human ethics permit no CF11/0766-2011000387) for this study was approved by the Monash University Human Research Ethics Committee (MUHREC). All adult volunteers provided informed written consent; no child participants were involved in the study.

## Supporting information

S1 Fig *w*Mel and *w*MelCS lines are bidirectionally compatible in *Ae*. *aegypti*. Bidirectional compatibility between *w*Mel and *w*MelCS lines was determined by crossing *Wolbachia*-infected females and males from each line, with control CI crosses performed between uninfected female mosquitoes (WT) and *Wolbachia-*infected males of each line. Bars are the mean percentage hatch rate ± SEM from 30 females (individual data points are superimposed).
(TIF)

S1 Methods Methods used to generate S1 Fig. are described in S1 Methods. (DOCX)

S1 Data All raw data are available within S1 Data. (XLSX)
