# Supplementary figures and images for: Novel Wolbachia-transinfected Aedes aegypti mosquitoes possess diverse fitness and vector competence phenotypes
Source: PLoS Pathog. 2017 Dec 7;13(12):e1006751. doi: 10.1371/journal.ppat.1006751 (PMC5736235; doi:10.1371/journal.ppat.1006751)

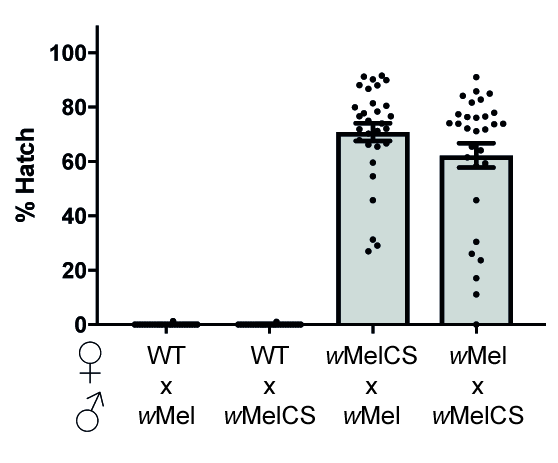

Supplement: S1 Fig — Bidirectional compatibility between wMel and wMelCS lines was determined by crossing Wolbachia-infected females and males from each line, with control CI crosses performed between uninfected female mosquitoes (WT) and Wolbachia-infected males of each line. Bars are the mean percentage hatch rate ± SEM from 30 females (individual data points are superimposed). (TIF) [file ppat.1006751.s001.tif]
