# Supplementary material for: Novel Wolbachia-transinfected Aedes aegypti mosquitoes possess diverse fitness and vector competence phenotypes
Source: PLoS Pathog. 2017 Dec 7;13(12):e1006751. doi: 10.1371/journal.ppat.1006751 (PMC5736235; doi:10.1371/journal.ppat.1006751)
Supplement: S1 Methods — (DOCX) [file ppat.1006751.s002.docx]

**Supplementary Methods.**

**Bidirectional Compatibility between *w*MelCS and *w*Mel**:

To investigate the bidirectional compatibility between *w*MelCS- and *w*Mel-infected *Ae. aegypti* the genetic background was first homogenized by backcrossing both strains into a highly inbred *Wolbachia*-free Rockefeller (Rock) background for six generations. The following four paired crosses were set up: (1) a *w*Mel compatibility test cross (*w*MelCS-Rock females x *w*Mel-Rock males), (2) a *w*MelCS compatibility test cross (*w*Mel-Rock females x *w*MelCS-Rock males), (3) a *w*Mel incompatibility cross (uninfected-Rock females x *w*Mel-Rock males), and (4) a *w*MelCS incompatibility cross (uninfected-Rock females x *w*MelCS-Rock males). Each cross consisted of a group of 50 virgin males and females (total 100) sexed at the pupae stage. In order to control for the age of the mosquitoes, adults emerging within 24 hours were used in the study. Each group was allowed to mate for 5 days after emergence. On day five all females were blood fed on the arms of one human volunteer. Gravid females were aspirated into individual tubes three days post blood feeding and allowed to oviposit on wet filter paper as described in the main text methods. Thirty egg-laying females were picked at random and used in hatch rate studies as described in the main text methods.
